# Isolation and characterization of GFAP-positive porcine neural stem/progenitor cells derived from a GFAP-CreER^T2^ transgenic piglet

**DOI:** 10.1186/s12917-018-1660-4

**Published:** 2018-11-07

**Authors:** Eunhye Kim, Seon-Ung Hwang, Junchul David Yoon, Hyunggee Kim, Gabsang Lee, Sang-Hwan Hyun

**Affiliations:** 1 Laboratory of Veterinary Embryology and Biotechnology, Veterinary Medical Center and College of Veterinary Medicine, Chungbuk National University, 1 Chungdae-ro, Seowon-gu, Cheongju, 28644 Republic of Korea; 2 Institute of Stem Cell & Regenerative Medicine (ISCRM), Chungbuk National University, Cheongju, 28644 Chungbuk Republic of Korea; 3 Department of Biotechnology, School of Life Sciences and Biotechnology, Korea University, 02841 Seoul, Republic of Korea; 4 Institute for Cell Engineering, Johns Hopkins University School of Medicine, Baltimore, MD USA

**Keywords:** Pig, Neural stem cells, Notch signaling, Reactive astrocytes

## Abstract

**Background:**

The porcine brain is gyrencephalic with similar gray and white matter composition and size more comparable to the human rather than the rodent brain; however, there is lack of information about neural progenitor cells derived from this model.

**Results:**

Here, we isolated GFAP-positive porcine neural stem cells (NSCs) from the brain explant of a transgenic piglet, with expression of CreER^T2^ under the control of the GFAP promoter (pGFAP-CreER^T2^). The isolated pGFAP-CreER^T2^ NSCs showed self-renewal and expression of representative NSC markers such as Nestin and Sox2. Pharmacological inhibition studies revealed that Notch1 signaling is necessary to maintain NSC identity, whereas serum treatment induced cell differentiation into reactive astrocytes and neurons.

**Conclusions:**

Collectively, these results indicate that GFAP promoter-driven porcine CreER^T2^ NSCs would be a useful tool to study neurogenesis of the porcine adult central nervous system and furthers our understanding of its potential clinical application in the future.

**Graphical abstract:**

ᅟ
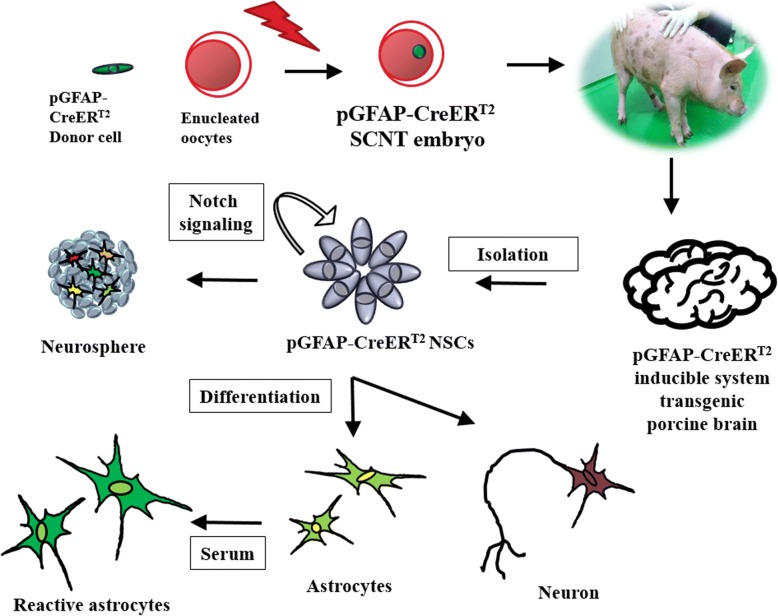

**Electronic supplementary material:**

The online version of this article (10.1186/s12917-018-1660-4) contains supplementary material, which is available to authorized users.

## Background

Neural stem cells (NSCs) in the mammalian central nervous system (CNS), which maintain neuro- and gliogenic capacity throughout adulthood, are only found in the subventricular zone (SVZ) of the lateral ventricles and the subgranular zone (SGZ) of the hippocampal dentate gyrus (DG) [1, 2]. Especially, within the SVZ, glial fibrillary acidic protein (GFAP)-positive type B1 cells are capable of multipotent differentiation and self-renewal in vitro [3–5], or are activated in vivo to differentiate into multiple types of progeny upon extrinsic stimuli by brain injury. Specifically, NSCs in the SVZ can differentiate into astrocytes or neuroblasts that migrate and integrate into the olfactory bulb circuitry [6]. SVZ type B1 NSCs have astroglial characteristics and express marker proteins such as GFAP; however, they can be distinguished from terminally differentiated, GFAP-expressing, adult cortical astrocytes on the basis of morphology, gene and protein expression profiles, and stem/progenitor cell properties, including their ability to produce neuronal cells [7, 8]. The discovery of GFAP-positive type B1 cells in adult mammals holds great promise for neurological regenerative medicine [9–11], as compared to primary cortical astrocytes, which are not as readily available; however, the signaling pathways and/or niche necessary for porcine NSC properties remain uncharacterized because of the lack of a suitable animal model.

The GFAP-CreER^T2^ transgenic mouse was developed in 2006 to examine GFAP-expressing cells in the context of primary astroglial cultures [12]. The fusion protein consists of a modified estrogen receptor ligand-binding domain and the Cre DNA recombinase (CreER^T2^) with expression driven by the GFAP promoter. Small animal models, such as rodents, have played a crucial role in regenerative medicine research, but are poor representations of the human nervous system. Alternatively, pigs are the non-primate animal species most closely related to human, and can be treated with surgery and anesthesia conditions nearly identical to those used in the clinic [13, 14].

Pigs have a gyrencephalic brain similar in size to that of humans and consistent gray and white matter composition [15, 16]. Little is known about porcine neural progenitor cells [17, 18]. As such, we isolated GFAP-positive NSCs from SVZ and neocortex explants of pGFAP- CreER^T2^ transgenic piglets [19]. The cells were then characterized for NSC expression markers and the capacity to form neurospheres and self-renewal. In addition, the effect of Notch pathway activation and serum treatment in pGFAP-CreER^T2^ NSCs neurogenic differentiation was investigated to determine the biology of GFAP-positive NSCs in the adult porcine brain.

## Results

### Isolation of primary cells from brain tissue explants of transgenic pGFAP-CreER^T2^ piglet

**Fig. 1:**
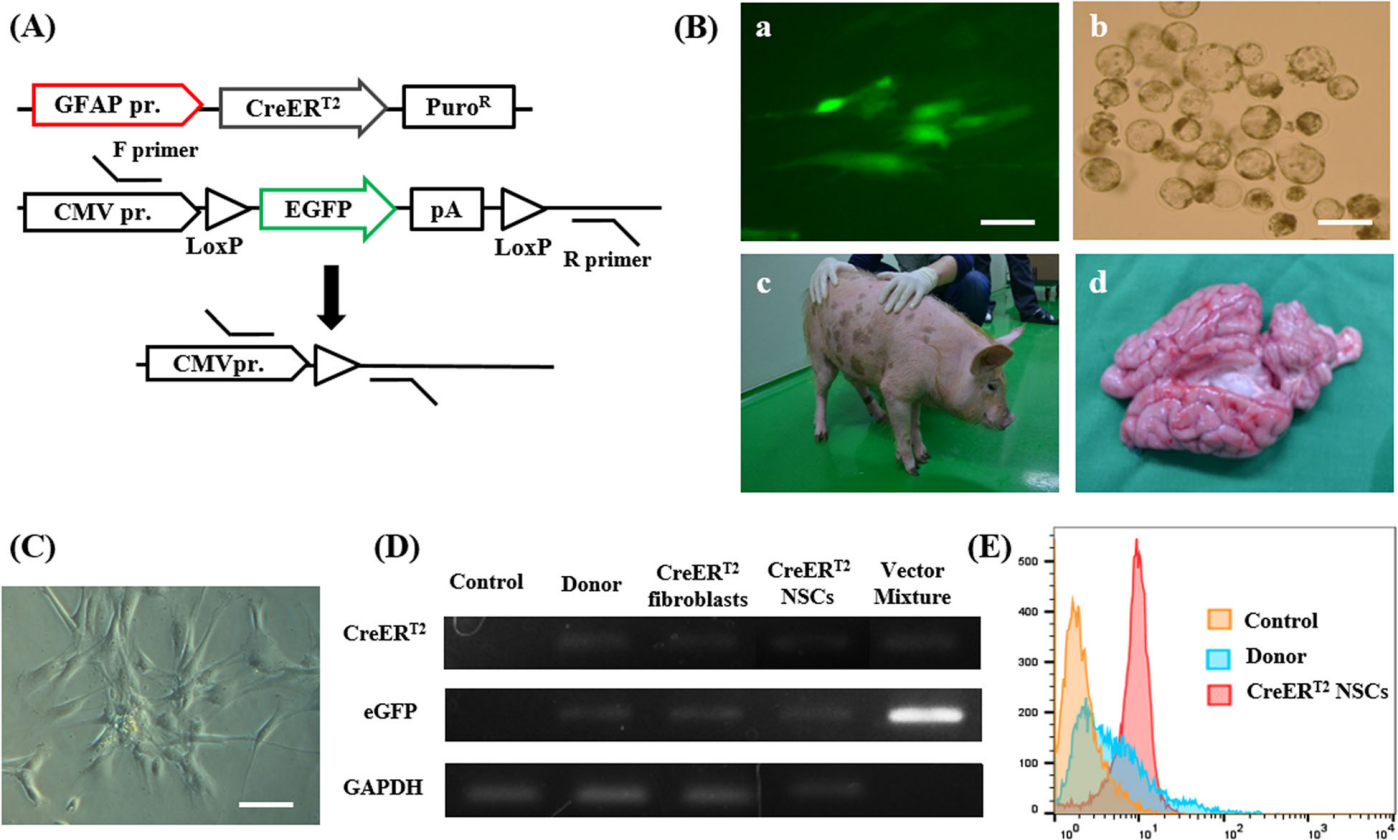
Isolation of primary cells from brain tissue explants of pGFAP-CreER^T2^ piglets. (A) Schematic of the vectors used in this experiment. (B) Representative image of pGFAP-CreER^T2^ fibroblast donor cells. Scale, 100 μm (a), pGFAP-CreER^T2^ nuclear transfer (NT) blastocysts. Scale, 200 μm (b), pGFAP-CreER^T2^ transgenic piglet (c), and brain organ (d), which were used to isolate neural stem cells (NSCs). (C) Representative brightfield images of primary pGFAP-CreER^T2^ NSCs. Scale, 50 μm. (D) Reverse transcriptase-PCR analysis was performed using genomic DNA isolated from the pGFAP-CreER^T2^ NSCs. (E) eGFP expression in pGFAP-CreER^T2^ NSCs was assessed by flow cytometry. Independent experiments were replicated at least three times

To achieve conditional GFAP gene expression, porcine fibroblasts were infected with a tamoxifen-inducible vector containing eGFP, which served as a source of nuclei for somatic cell nuclear transfer (SCNT) (Fig. 1A, Additional file 1: Figure S1). We subsequently harvested brain tissue from the generated pGFAP-CreER^T2^ transgenic piglets [19] (Fig. 1B). Notably, cultured cells isolated from the neocortex and SVZ exhibited a radial morphology (Fig. 1C). Data from genomic DNA analysis in these cells confirmed the integration of each transgene (CreER^T2^ and eGFP) into these cells (Fig. 1D). Subsequent flow cytometry analysis confirmed that the pGFAP-CreER^T2^ NSC population was homogenous for low eGFP expression as compared to donor cells (Fig. 1E).

### Porcine pGFAP-CreER^T2^ GFAP-positive cells express NSC-related proteins and form neurospheres in vitro

**Fig. 2:**
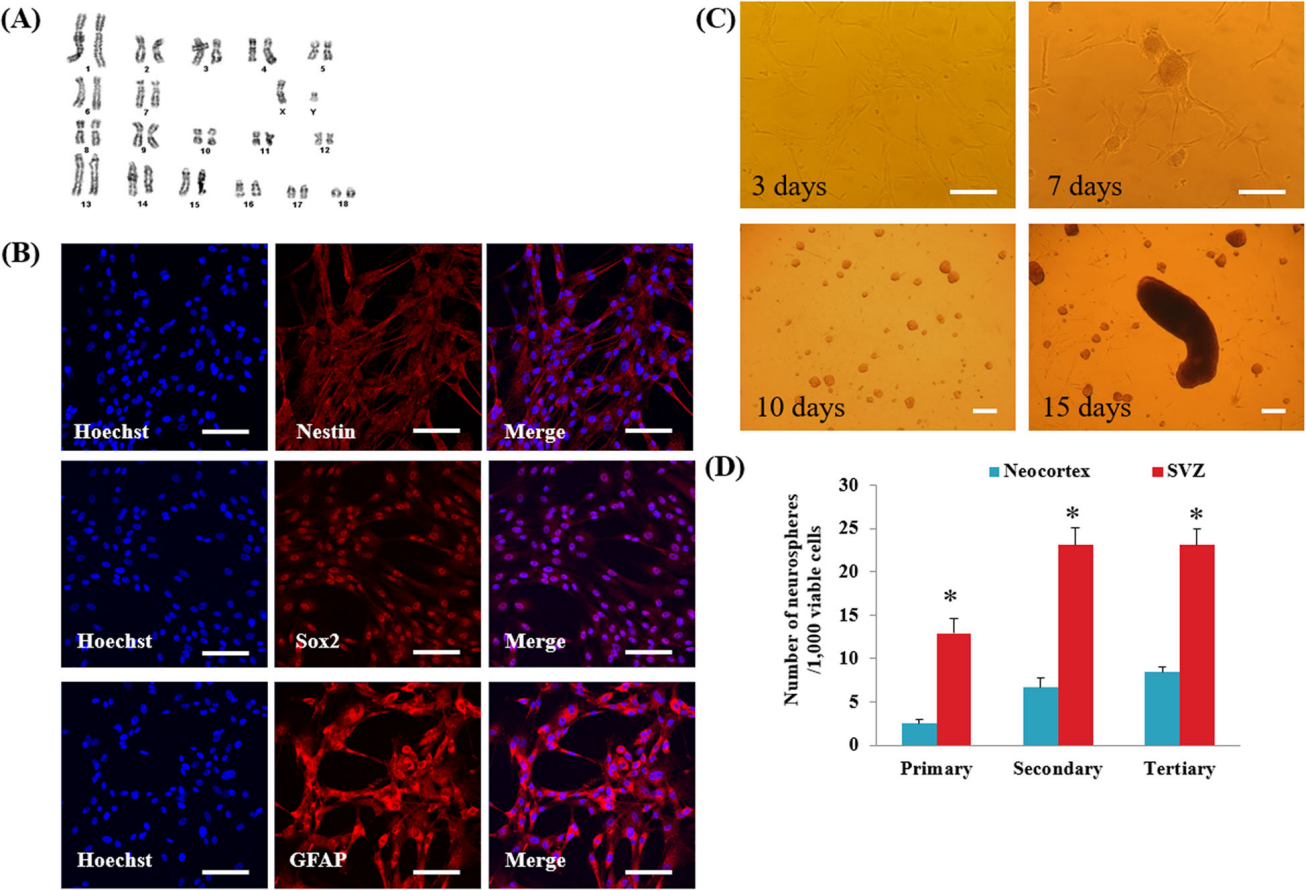
Characterization of porcine pGFAP-CreER^T2^ NSCs. **a** Karyotype analysis of pGFAP-CreER^T2^ NSCs. **b** Immunofluorescence analysis of the neural stem cell markers Nestin, Sox2, and GFAP in pGFAP-CreER^T2^ NSCs. Scale, 100 μm. **c** Primary neurosphere-generating cells from adult porcine pGFAP-CreER^T2^ brain tissue cultured in the presence of EGF and FGF2. Scale, 100 μm. **d** Quantification analysis of primary, secondary, and tertiary sphere formation from cells obtained from adult porcine pGFAP-CreER^T2^ neocortex or SVZ. Neurosphere number was determined 10 days after plating. Data on the neurosphere number are the average ± SEM of 4 independent experiments consisting of 3 replicates. *P < 0.05

The isolated pGFAP-CreER^T2^ NSCs showed normal 36 + XY karyotype, and no discernable cytogenetic abnormalities at passage 7 (Fig. 2a). Immunofluorescence analysis confirmed homogenous cytoplasmic and nuclear expression of the NSC markers Nestin and Sox2, respectively (Fig. 2b). In comparison, GFAP expression was homogenous and intensive, as shown in Fig. 2b, whereas the cells were devoid of oligodendrocyte MBP and neuronal TUJ1 (data not shown). In the presence of EGF and FGF2, pGFAP-CreER^T2^ primary cultures formed plated spherical cell masses at 7 days after plating (Fig. 2c). Additionally, floating neurospheres were generated from these cell masses with morphology and size distribution similar to that of porcine neurospheres reported by previous studies, indicative of a self-renewal capacity [18]. After 15 days, we also observed giant spheres 600 μm in diameter, which were thought to be formed by the aggregation of individual spheres. When these spheres were dissociated and passaged, we also obtained novel “secondary” spheres that were morphologically similar to the primary spheres. Replating dissociated cells with second passage led to tertiary spheres with a similar morphology but diminished growth rate compared to that of secondary spheres (data not shown). Quantitative analysis demonstrated a yield of 2.50 ± 0.44 and 12.92 ± 1.67 primary spheres per 1000 viable cells from the neocortex and SVZ, respectively, whereas 6.67 ± 1.10 and 23.08 ± 1.96 secondary spheres were obtained from 1000 viable cells 10 days after plating from the same regions (Fig. 2d). Tertiary spheres (8.42 ± 0.99 spheres versus 23.08 ± 1.91 spheres, from neocortex and SVZ cells, respectively) could also be obtained from 1000 viable cells 10 days after a next passage, indicating that the cells continued to proliferate in vitro. Collectively, these results suggest that the isolated primary cells displayed a phenotype representative of GFAP-positive type B1 NSCs, and thus, were referred to as pGFAP-CreER^T2^ NSCs in subsequent experiments.

### Control of porcine pGFAP-CreER^T2^-NSC self-renewal by Notch1-dependant signaling

**Fig. 3:**
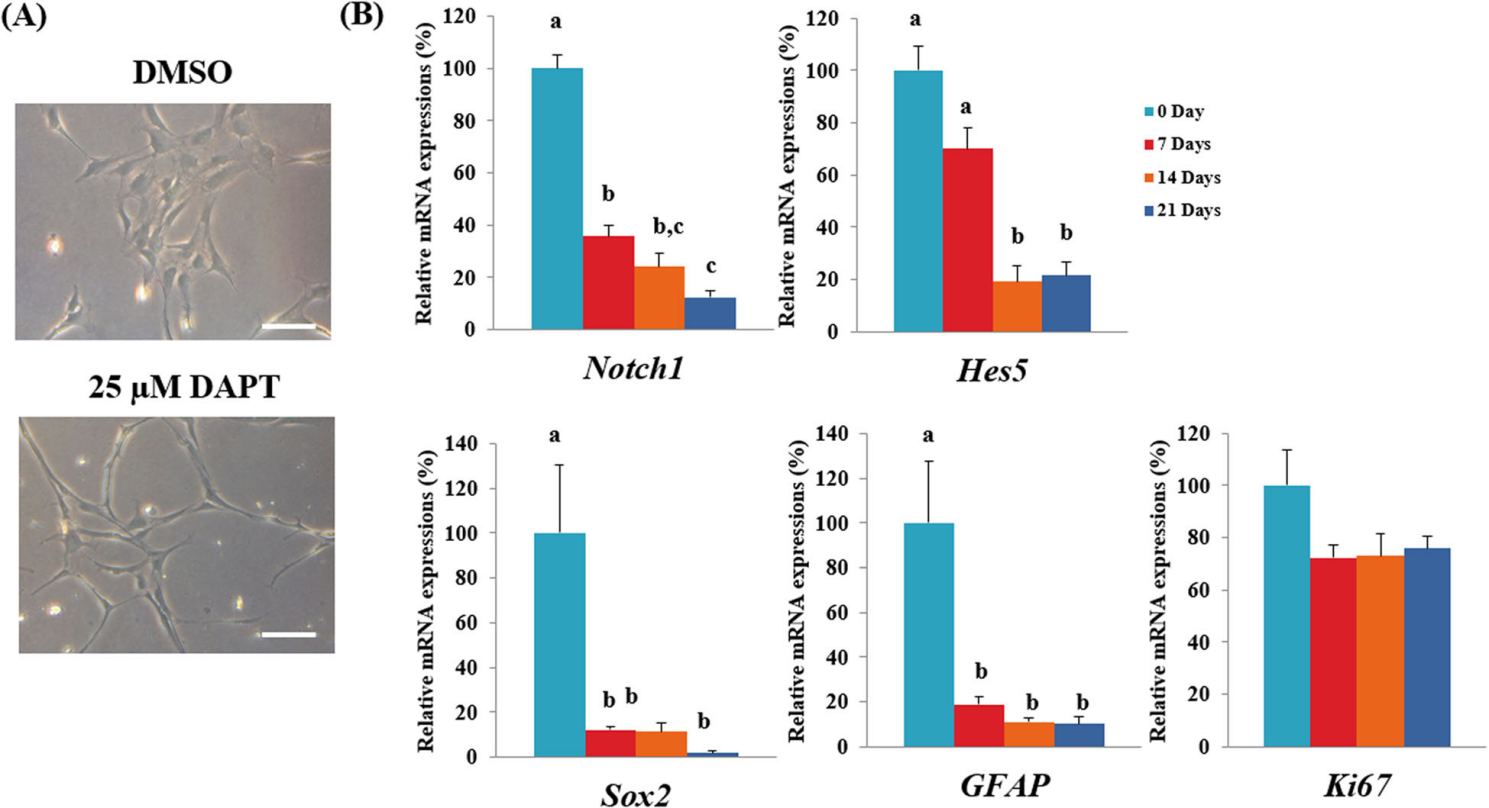
Effect of the Notch inhibitor DAPT on porcine pGFAP-CreER^T2^ NSCs. (A) Phase contrast image of pGFAP-CreER^T2^ NSCs with or without of 25 μM DAPT treatment. (B) qRT–PCR analysis of *Notch1, Hes5, Sox2, Gfap,* and *Ki67* in 25 μM DAPT treated pGFAP-CreER^T2^ NSCs. Bars with different letters (a-c) indicate a statistically significant difference between groups (*P < 0.05*). Independent experiments were replicated at least three times

To determine the necessity of Notch1 signaling in pGFAP-CreER^T2^ NSC maintenance, cells were cultured in the presence of the γ-secretase inhibitor DAPT (*N*-[*N*-(3,5-difluorophenacetyl)-l-alanyl]-sphenylglycine *t*-butyl ester), which blocks pathway activity. Notably, DAPT treatment induced a morphological change in pGFAP-CreER^T2^ NSCs, from a fusiform to a stellated phenotype (Fig. 3A). As expected, qRT-PCR analysis clearly showed decreased *Notch1* expression after 1 week of DAPT treatment (Fig. 3B). Moreover, the expression of Sox2, GFAP, and Hes5—a key target gene and effector of the Notch pathway—also declined after DAPT treatment, suggesting a correlation between these factors. Thus, we concluded that γ-secretase activity plays an essential role in maintenance of the GFAP-positive pGFAP-CreER^T2^ NSCs phenotype owing to its dependency on Notch1 signaling. In contrast, there is only a tendency of lower expression in *Ki67* at 7 days after DAPT treatment but no significant differences were observed indicating that 25 μM DAPT may not differentiated the cells to the level of affecting proliferation ability.

### Spontaneous differentiation of porcine pGFAP-CreER^T2^ NSCs

**Fig. 4:**
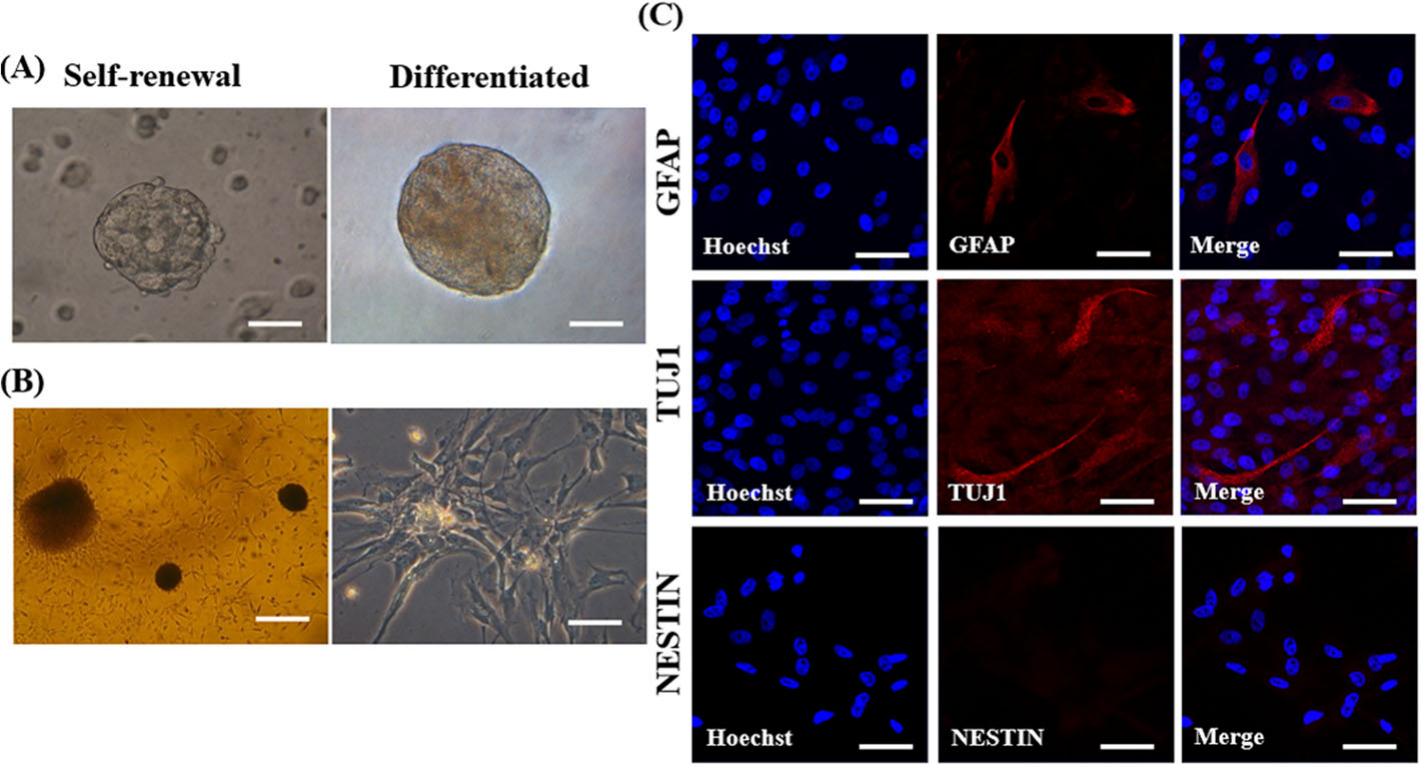
Spontaneous differentiated cells from porcine pGFAP-CreER^T2^ NSCs. **a** Brightfield image of pGFAP-CreER^T2^ neurospheres cultured in maintenance or differentiation media for 10 days. Scale, 200 μm. **b** Representative image of the formation neural network (left). Phase contrast image of differentiated pGFAP-CreER^T2^ cells at a higher magnification (right). Scale, 50 μm **c** Immunofluorescence analysis for astrocyte GFAP, neuronal TUJ1, and NSC Nestin marker expression in differentiated cells at 2 weeks. Scale, 50 μm

To assess multipotency and lineage commitment, pGFAP-CreER^T2^ NSCs derived neurospheres were spontaneously differentiated using FGF2- and EGF-depleted medium for 10 days (Fig. 4a). After replating, the differentiated neurospheres adhered to the culture plastic and migrated to the outside of the dish in the mitogenic factors-depleted medium (Fig. 4b). The cells at the sphere periphery formed contacts with other neurospheres to construct a cellular network. Also, the differentiated cells lost both Nestin expression with decreased GFAP expressing cells (Fig. 4c), but some showed increased neuronal TUJ1 expression (Fig. 4c); however, the cells were devoid of SOX2 and oligodendrocyte O4 (data not shown). This results suggests that the pGFAP-CreER^T2^ NSCs were also shown to spontaneously differentiate in vitro.

### Effect of serum treatment on porcine pGFAP-CreER^T2^ NSC-derived astrocytes

**Fig. 5:**
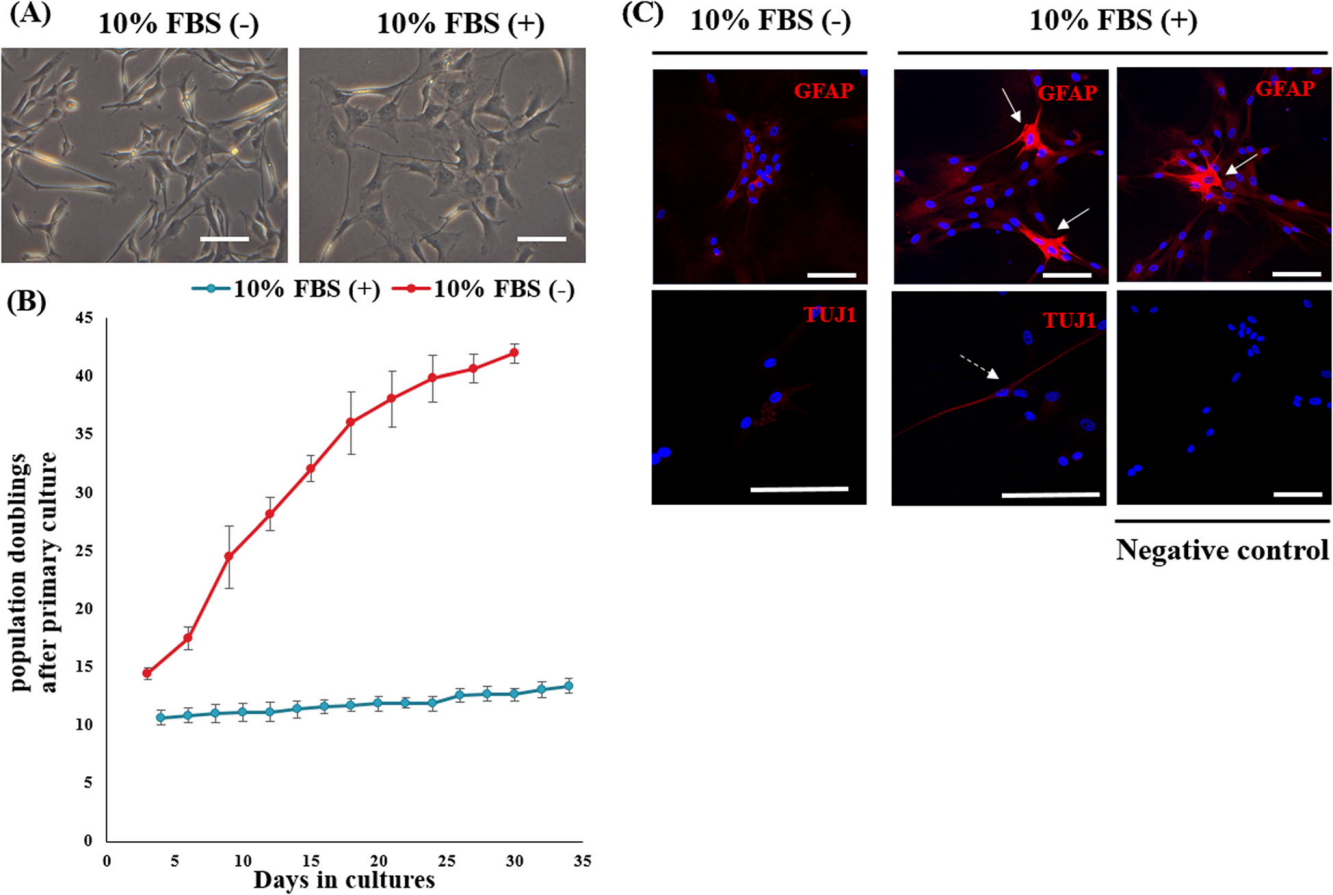
Effect of serum treatment on porcine pGFAP-CreER^T2^ NSCs. **a** Phase contrast image of porcine pGFAP-CreER^T2^ NSCs with or without 10% FBS treatment. Scale, 50 μm. **b** Total cell population doublings estimated by cell counting at each passage. Independent experiments were replicated three times. **c** Representative confocal images showing the expression of astrocytic GFAP and neuronal TUJ1 after differentiation. Some cells revealed a strong GFAP expression (white arrow) or TUJ1 expression (dashed white arrow) in FBS treated groups. Scale, 100 μm

To determine if pGFAP-CreER^T2^ NSC-derived astrocytes could respond to reactive cues such as serum, cells were cultured in astrocytic differentiation medium for 45 days. Notably, in the absence of serum, the GFAP-positive astrocytes formed a monolayer with abundant thin projections, but adopted a reactive phenotype with polygonal morphology upon treatment with 10% FBS (Fig. 5a). We also monitored population doublings (PD) by counting individual cells from passages 1 to 15 and found that the 10% FBS-treated group showed increased rate of proliferation compared to the 10% FBS-untreated group, as expected (Fig. 5b). As shown in Fig. 5c, all of the astrocytes showed the homogeneously decreased GFAP expression in the absence of serum; however, some FBS-treated cells (white arrows) exhibited strong GFAP protein expression with stellation, which is the clear indicative of reactive astrogliosis. We also observed TUJ1 expression in some of the differentiated cells, which displayed a mature morphology with neuronal projections, in the FBS-treated group. Collectively, these data demonstrate that serum treatment can induce a reactive phenotype and neuronal maturation of pGFAP-CreER^T2^ NSC-derived astrocytes in vitro.

## Discussion

In this study, we isolated and characterized pGFAP-CreER^T2^-NSCs from adult transgenic piglet. Consistent with other NSC studies, the isolated pGFAP-CreER^T2^ NSCs displayed the normal NSC phenotype, such as Notch-dependent self-renewal, and differentiation capacity to neurons and reactive astrocytes.

Quiescent and activated stem cells coexist in the adult stem cell niche [20], and play critical roles in maintenance, regeneration, function, aging, and disease. Our observations with pGFAP-CreER^T2^ NSCs raise questions on the activation status of NSCs used in these previous studies. A recent study concluded that quiescent adult NSCs can be identified by coexpression of the markers CD133, GFAP, and EGFR [21]. Their results showed that quiescent NSCs lack expression of the hallmark NSC marker Nestin [22], which is only upregulated upon cell activation. All of the pGFAP-CreER^T2^ NSCs examined in our study expressed Nestin and Sox2, suggesting that the isolated NSCs likely activated in response to culturing in vitro. Since NSCs progress through multiple intermediate states during activation [23], the identification of stage-specific markers will be important to optimize the isolation and culture methods reflected in this study.

Adult neurogenesis is observed in all vertebrate species [24], and NSCs have previously been isolated from the CNS of adult rodents [25] and humans [26]. These neural progenitors share largely common isolation methods and defined in vitro culture conditions [27]. Progenitor cells can be cultured as either monolayers or floating spherical masses of undifferentiated cells, termed as neurospheres [11, 28], the latter of which demonstrates capacities for proliferation, self-renewal, and differentiation. Notably, cells derived from the porcine CreER^T2^ neocortex and SVZ formed neurospheres, although the SVZ showed a greater sphere-forming potential.

Strong evidence suggests that adult GFAP-positive SVZ NSCs are always in contact with the ventricular cerebrospinal fluid (CSF) via apical process with a primary cilium that protrudes through the ependymal cells, as well as a basal process that terminates on a blood vessel [29, 30]. A number of extracellular signals in the SVZ niche—including FGF2 and EGF—have been shown to act as critical factors to maintain NSC self-renewal [31]. Without these mitogenic growth factors, pGFAP-CreER^T2^ NSCs undergo spontaneous differentiation characterized by Nestin downregulation and an increase in TUJ1 expression in some cells. In addition, LIF was used for NSC maintenance in the present protocol as compared to that in other studies on porcine neural precursor cells, which used EGF and bFGF. LIF promotes astrocyte differentiation via JAK/STAT and MAPK pathway activation [32]; however, it has been shown to block the neuronal differentiation of human NSCs, and prevent reactive oxygen species accumulation and apoptosis by caspase-3 and -7 inhibition, resulting in enhanced cell proliferation and self-renewal [33, 34]. No other small molecules were needed for pGFAP-CreER^T2^ NSCs maintenance.

Notch signaling is a pivotal regulator of quiescence and causes heterogeneity in NSCs with respect to the activated or quiescent state, morphology, gene expression profile, and response to injury [35–37]; it is involved in the maintenance of the undifferentiated NSC state [38, 39] and cell fate decision inducing gliogenesis or neurogenesis [40–42] in a stage-dependent manner. NSC maintenance requires γ-secretase activity and Notch activity, which acts by altering *Hes5* expression [43, 44]. As expected, our results showed that NSC identity declined with DAPT treatment, suggesting that Notch signaling plays similar roles in the human and porcine SVZ niche.

It should be noted that some limitations are associated with the long-term culture of pGFAP-CreER^T2^ NSC-derived neurospheres, as previously reported in humans [45, 46]. For instance, cells became less proliferative with prolonged culture. FBS treatment can enhance proliferation, but concurrently incites differentiation. In this study, the pGFAP-CreER^T2^-NSC-derived astrocytes proliferated in normal astrocyte culture medium without any additional factors other than 10% FBS, similar to that observed with human NSCs [34]. Understanding of the mechanism mediating NSC maintenance in the SVZ niche is critical to brain function, both under normal conditions or after cortical injury. Astrocytes undergo “reactive gliosis” in response to many CNS pathologies—such as trauma, tumor, or neurodegenerative disease, which is characterized by hypertrophy and a marked increase in GFAP expression [47, 48]. Our results revealed that serum induced reactive gliosis in pGFAP-CreER^T2^ NSC-derived astrocytes, consistent with the possibility of serum as a potent activator of reactive astrogliosis. There is a growing awareness of heterogeneity among multiple levels of reactive astrocytes [49] characterized by canonical features [50–52]. Since the pGFAP-CreER^T2^-NSCs were generated from the same animal, these NSCs would be a cell source to study porcine neurogenesis.

## Conclusions

In the present study, we obtained activated pGFAP-CreER^T2^ NSCs with a protoplasmic morphology and low GFAP expression—which may be attributed to CMV promoter methylation—as well as induced reactive gliosis in cells resulting in stellate morphology with a hypertrophic cell soma and processes, pronounced GFAP expression, and connections with neighboring astrocyte processes. The most important finding was the necessity of Notch signaling for pGFAP-CreER^T2^ NSC maintenance. While the functional significance of porcine NSCs to neurogenesis in adult porcine brain remains unclear, the present study provides further understanding on the role of GFAP-positive progenitor cell dynamics in adult porcine neurogenesis in vitro.

## Methods

### Chemicals

All chemicals were purchased from Sigma-Aldrich (St. Louis, MO, USA) unless stated otherwise.

### Isolation and culture of pGFAP-CreER^T2^ NSCs

In our previous study, we produced and reported pGFAP-CreER^T2^ piglet [19]. We excised whole brains from 4-month-old pGFAP-CreER^T2^ piglet immediately after sacrifice, placed in 2 mL fresh Hank’s balanced buffered saline (HBSS), and dissected under a stereomicroscope. First, the olfactory bulb and cerebellum were removed with fine dissecting forceps and a midline incision was performed between the hemispheres. The meninges was then pulled, using fine forceps, and removed from the cortex hemisphere. The brain tissue was then dissected into two parts, neocortex and SVZ, minced, and transferred into a sterile 50-m: Falcon tube filled containing 22.5 mL HBSS and 2.5 mL of 2.5% trypsin. The conical was incubated in a 37 °C water bath for 30 min with gentle shaking every 10 min. The resulting suspension was centrifuged and the pellet dissociated into single cells with vigorous pipetting in 10 mL porcine NSC medium. The mixture was then plated in 6-well plates coated with 2 mL of 50 μg/mL poly-d-lysine (PDL) for 1 h at 37 °C. The porcine NSC medium was composed of DMEM/F10 medium (Gibco) supplemented with 40 ng/mL EGF (Peprotech), 40 ng/mL bFGF (Peprotech), N2 supplement (Invitrogen), B27 supplement (Invitrogen), glutamine (Invitrogen), penicillin/streptomycin and 1.5 ng/mL leukemia inhibitory factor (LIF, ESGRO, Chemicon, Temecula, CA, USA). The medium was changed 2 days after plating and everyday thereafter. For passage, the primary spheres from each explant were collected in sterile 15 mL conical tubes, incubated for 45 min at 37 °C in 200 μL TrypLE™ Express solution (Gibco Invitrogen) per 5 mL culture, and then gently dissociated gently with a 26-ga needle attached to a disposable syringe. The dispersed cells were resuspended in fresh culture medium and replaced with 2 mL per well of fresh medium every 2 days. Then, porcine NSCs at 70–80% confluency were split at a 1:3 ratio with accutase.

### Flow cytometry

Cells (500,000 cells/mL) were suspended in 0.5 mL FACs buffer consisting of dPBS with 1% FBS and analyzed with a FACs Aria II (BD Immunocytometry Systems, San Jose, CA, USA) and FlowJo software (Tree Star, Ashland, OR, USA).

### Karyotyping

The porcine pGFAP-CreER^T2^ NSCs were induced to metaphase by 10 μg/mL colcemid (Gibco, Carlsbad, CA, USA) for 4–5 h. Then, the karyotyping analysis was performed as previously described [53].

### Gene expression analysis by real-time PCR or reverse transcription PCR

pGFAP-CreER^T2^ NSCs or differentiated cells were analyzed for the expression of neural and progenitor markers by RT-PCR or comparative qRT-PCR. The groups of cells were harvested separately and stored at − 80 °C until use. The analysis was performed as previously described [53], except that genomic DNA was isolated from cells using G-spin™ Genomic DNA Extraction kit buffer (iNtRON Biotechnology, SungNam, Korea) and transgenes in genomic DNA were amplified with the appropriate primer set (EGFP, CreER^T2^, or GAPDH). Also, the housekeeping gene *RN18S* served as an internal control to rule out the possibility of RNA degradation and differences in mRNA concentration and qRT-PCR was carried out by 35 cycles of denaturation at 95 °C for 30 s, annealing at 55 °C for 30 s, and extension at 72 °C for 30 s. All oligonucleotide primer sequences are found in Table 1. The fluorescence intensity was measured at the end of the extension phase of each cycle with threshold values set manually. Relative expression was determined by the 2^ΔΔCt^ method, with *RN18S* as a control. Experiments were replicated at least three times.

**Table 1:** Porcine specific primers used for gene expression analysis

Genes	Primer sequences	Products
		size (bp)
*CreER* ^*T2*^	5’-TCGCAAGAACCTGATGGACA-3′	288
	5’-CGCCGCATAACCAGTGAAAC-3′	
*eGFP*	5’-AACGGCCACAAGTTCAGCGT-3′	424
	5′- TCACCTTGATGCCGTTCTTC -3’	
*Notch1*	5’-TGGATGGCATCAATTCCTTT-3’	138
	5′- ACTTGTAGGCGCCATAGCTG-3’	
*Hes5*	5’-TTCTCAGAGAATGTGTGTGCAGAGT-3’	75
	5’-GGTCAGACACTTGGCAGAAGATG-3’	
*Sox2*	5’-CGGCGGCAGGATCGGC-3’	113
	5’-GAGCTCCGCGAGGAAAA-3’	
*GFAP*	5’-CACAAGGATGTGGTGTGAGG-3’	207
	5’-TGCTACCTGGCAGGCTTAAT-3’	
*Ki67*	5’-GAAACCCAGATCCGAGCATA-3’	139
	5’-CAGCAGCTATTCTGGCAACA-3’	
*GAPDH*	5’-GTCGGTTGTGGATCTGACCT-3’	207
	5’-TTGACGAAGTGGTCGTTGAG-3’	
*RN18S*	5’-CGCGGTTCTATTTTGTTGGT-3’	219
	5’-AGTCGGCATCGTTTATGGTC-3’	

### Immunofluorescence (IF) analysis

The IF analysis was performed as previously described [53]. Briefly, fixed and washed cells were permeabilized with 0.2% Triton X-100 for 5 min for intracellular markers analysis and co-incubated with blocking solution (10% goat serum in PBS) and primary antibody overnight at 4 °C. The primary antibodies used in this study are found in Table 2. After washing, cells were incubated with appropriate secondary antibodies at room temperature for 1 h. After the nuclei were stained with 10 μg/ml Hoechst 33342 (B2261), cells were mounted with anti-fade reagent (S36937, Invitrogen) and examined under confocal microscope (LSM700, Carl Zeiss, Oberkochen, Germany) and ZEN 2009 Light Edition software (Carl Zeiss, Oberkochen, Germany).

**Table 2:** Antibodies used for immunofluorescence staining

Detection of	Name	Host species	Dilution	Manufacturer
NPC^a^	NESTIN	Mouse IgG	1:500	Millipore MAB5326
ESC^b^ / NPC	SOX2	Rabbit IgG	1:200	Millipore AB5603
Astrocytes / Radial glia	GFAP	Rabbit IgG	1:1000	Millipore PAB5804
Young neurons	TUJI	Mouse IgG	1:200	Millipore MAB1637

^a^Neural progenitor cell, ^b^Embryonic stem cell

### Differentiation of pGFAP-CreER^T2^-NSCs

For spontaneous NSC differentiation, neurospheres were seeded in wells containing poly-d-lysine (Sigma), using a Pasteur pipette, and cultured in the absence of EGF and bFGF for 10 days. To differentiate NSCs into astrocytes, after reaching 70–80% confluency, the NSC culture medium was replaced with astrocyte differentiation medium consisting of N2B27 media supplemented with 1% FBS (Gibco).

### Statistical analysis

Statistical analysis was performed using SPSS 17.0 (SPSS, Inc., Chicago, IL, USA). Results are expressed as the means ± SEM. One-way ANOVA was performed to test the null hypothesis of group differences, followed by Duncan’s multiple range test or Student’s t-test. *P* < 0.05 was considered statistically significant.

## Additional file

Additional file 1: **Figure S1**. Generation of pGFAP-CreER^T2^ embryos using pGFAP-CreER^T2^ fibroblasts as donor cells. Scale bars = 50 μm. (TIF 1443 kb)

## Abbreviation

ANOVA: Analysis of variance
cDNA: Complementary DNA
CNS: Central nervous system
CreER^T2^: Cre DNA recombinase
CSF: Cerebrospinal fluid
CT: Threshold cycle
DAPT: N-[N-(3,5-difluorophenacetyl)-L-alanyl]-sphenylglycine t-butyl ester
DG: Dentate gyrus
DPBS: Dulbecco’s phosphate buffered saline
EGF: Epidermal growth factor
eGFP: Enhanced green fluorescent protein
ESC: Embryonic stem cells
FBS: Fetal bovine serum
FGF: Fibroblast growth factor
GAPDH: Glyceraldehyde 3-phosphate dehydrogenase
GFAP: Glial fibrillary acidic protein
HBSS: Hank’s Balanced Salt Solution
LIF: Leukemia inhibitory factor
mRNA: Messenger RNA
NPC: Neural progenitor cell
NSC: Neural stem cell
NT: Nuclear transfer
PDL: Poly-D-lysine
PD: Population doublings
pGFAP-CreER^T2^: CreER^T2^ under the control of the porcine GFAP promoter
RT-PCR: Reverse transcription polymerase chain reaction
qRT-PCR: Quantitative real-time polymerase chain reaction
RN18S: 18S ribosomal RNA
SCNT: Somatic cell nuclear transfer
SEM: Standard error of mean
SPSS: Stepwise discriminant analysis
SVZ: Subventricular zone
