# Supplementary figures and images for: Isolation and characterization of GFAP-positive porcine neural stem/progenitor cells derived from a GFAP-CreERT2 transgenic piglet
Source: BMC Vet Res. 2018 Nov 7;14:331. doi: 10.1186/s12917-018-1660-4 (PMC6222979; doi:10.1186/s12917-018-1660-4)

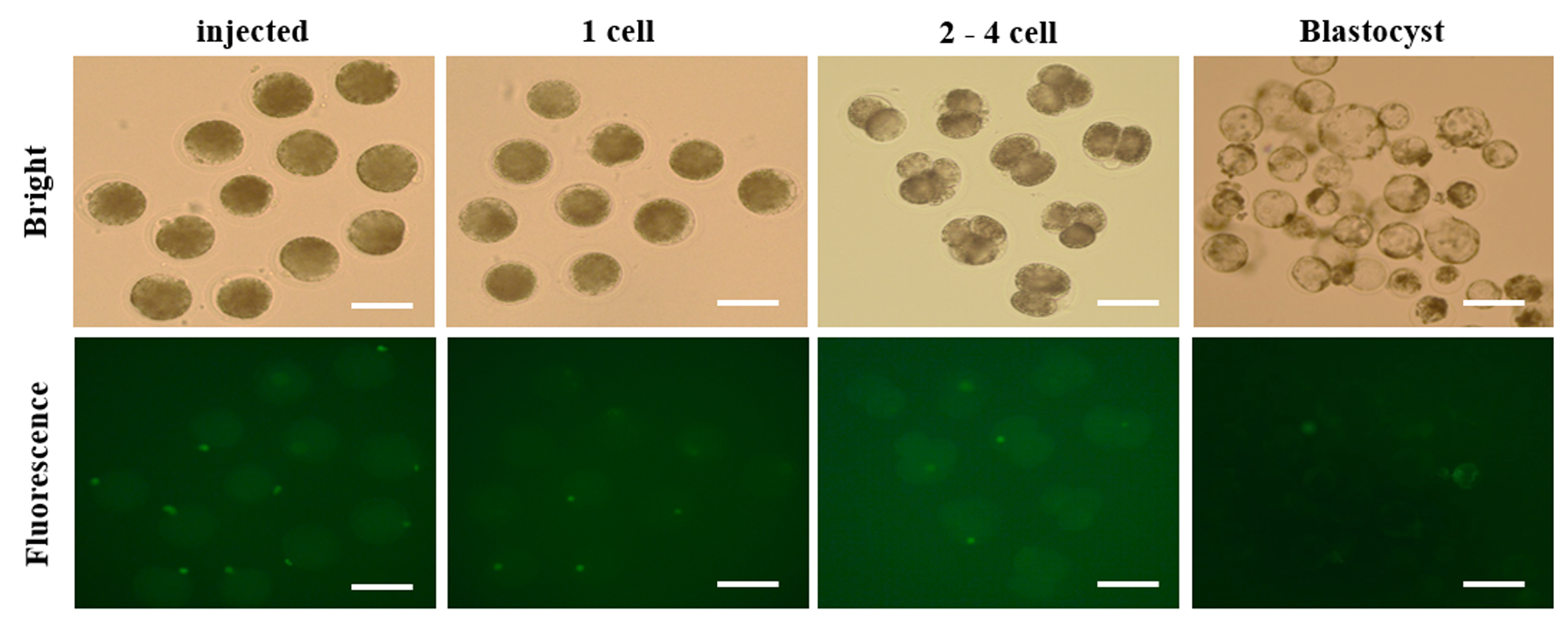

Supplement: Supplementary file 1 — Figure S1. Generation of pGFAP-CreERT2 embryos using pGFAP-CreERT2 fibroblasts as donor cells. Scale bars = 50 μm. (TIF 1443 kb) [file 12917_2018_1660_MOESM1_ESM.tif]
